# Effectiveness of an Evidence-Based Practice Education Program for Undergraduate Nursing Students: A Systematic Review and Meta-Analysis

**DOI:** 10.3390/ijerph21050637

**Published:** 2024-05-16

**Authors:** Dain Jeong, Chang Park, Keiko Sugimoto, Miyang Jeon, Dooyoung Kim, Young Eun

**Affiliations:** 1 College of Nursing, University of Illinois Chicago, Chicago, IL 60612, USA; djeong22@uic.edu (D.J.); ksugim2@uic.edu (K.S.); 2 Department of Population Health Nursing Science, College of Nursing, University of Illinois Chicago, Chicago, IL 60612, USA; parkcg@uic.edu; 3 College of Nursing, Institute of Medical Sciences, Gyeongsang National University, Jinju 52727, Republic of Korea; myjeon68@gnu.ac.kr; 4 College of Nursing, Gyeongsang National University, Jinju 52727, Republic of Korea; kdy83@gnu.ac.kr

**Keywords:** evidence-based practice, evidence-based nursing, nursing education, competency, critical thinking, problem-solving ability

## Abstract

This systematic review and meta-analysis examined the impact of evidence-based practice (EBP) education programs on undergraduate nursing students, focusing on enhancing EBP competency, critical thinking, and problem-solving ability. Methods: The search, conducted through PubMed, Cochrane Library, EMBASE, CINAHL, and Web of Science up to December 2023, included studies published in English and Korean and adhered to PRISMA guidelines. Qualitative appraisal of the studies was conducted using the revised ROB II for randomized trials and the ROBINS-I for non-randomized trials. For the meta-analysis, the effect size of the intervention was calculated as a standardized mean difference. Results: In our study, 11 studies met our inclusion criteria, and 8 studies of those were included in the meta-analysis. The effect sizes for EBP competency, critical thinking, and problem-solving ability were 1.55, 1.29, and 0.65, respectively. The meta-regression analysis indicated that tailored education programs of 4–7 weeks and being in the 4th grade significantly enhanced EBP competency. Conclusion: These findings support the development of a customizable and applied EBP education actively for students, preparing nursing students to effectively implement EBP in clinical settings after graduation. Despite the significant effect size of the outcome variables, the high heterogeneity suggests the need for further investigation to validate the EBP educational outcomes for nursing students.

## 1. Introduction

Evidence-based practice (EBP) aims to optimize clinical outcomes by integrating the highest-quality evidence from systematic research with the clinical expertise of nurses, along with patient preferences and available resources [1]. The International Council of Nurses stresses the importance of clinical nurses actively participating in research and applying their findings to enhance EBP [2]. Generally, EBP promotes safer patient care, improves outcomes, reduces the time spent on nursing care, decreases medical costs, and minimizes regional disparities in services through standardized practices. It also boosts nurses’ competence and job satisfaction, ultimately benefiting patients, the nurses themselves, and the overall healthcare system [3].

Li et al. [4] discovered that, although nurses had positive attitudes towards EBP, they often lack the necessary knowledge and skills. Furthermore, it was noted that nurses are inadequately trained in applying EBP and seldom implement it in the field [5]. Melnyk et al. [6] highlighted that the level of education is closely related to the implementation of 

EBP, indicating that an undergraduate education not only raises awareness of these practices but also fosters a readiness to employ EBP in clinical practice. Therefore, substantial education and training are essential to develop the skills to find the best evidence and integrate it with nurses’ expertise and patient preferences [1].

To effectively implement EBP, various elements must be considered. EBP competency is defined as the ability to ask clinically relevant questions within the context of care, which involves the acquisition, evaluation, application, and reevaluation of various knowledge sources [7]. Additionally, critical thinking is essential for skilled nursing interventions; it involves actively and skillfully recognizing, analyzing, synthesizing, and evaluating collected information [8]. Furthermore, problem-solving ability is crucial for EBP, as it enables nurses to efficiently resolve clinical issues and implement EBP interventions [9]. Given these factors, it is important to cultivate these competencies and integrate EBP education into the undergraduate nursing curriculum [10].

When examining previous studies on the effectiveness of EBP education programs, research had been conducted across various populations, including undergraduate students, registered nurses advancing to a Bachelor of Science in nursing [11], health professionals [12,13], and nurses [14]. These studies employed online, face-to-face, or blended learning modalities and varied widely in duration—from as brief as 30 min to an entire semester. The content of these EBP programs ranged from a partial to complete coverage of the EBP steps and utilized methodologies such as lectures, workshops, discussions, and practicums or a mix of these approaches. Meta-analyses focusing on nurses’ education in EBP [14] have demonstrated significant effects on knowledge, skills, attitudes, and behaviors related to EBP.

However, systematic analyses specifically targeting EBP education programs for undergraduate nursing students are limited, thereby restricting the availability of crucial information applicable in educational contexts. Therefore, this study aims to systematically review the literature concerning the effectiveness of EBP education programs for undergraduate nursing students, perform a meta-analysis to estimate a comprehensive effect size, and provide foundational evidence to support the future development of effective EBP education programs for undergraduate nursing students.

## 2. Methods

### 2.1. Study Design

This study involved a systematic review and meta-analysis to assess the impact of EBP education on the outcomes of undergraduate nursing students. It adhered to the Preferred Reporting Items for Systematic Reviews and Meta-Analyses (PRISMA) guidelines [15] (see Supplementary Table S1) and was registered in the PROSPERO database (CRD 42024512947).

### 2.2. PICO Framework

For the systematic review, our inclusion criteria were based on the PICO-SD (Participants, Intervention, Comparison, Outcome, and Study design) as follows: studies of undergraduate students enrolled in nursing courses (participants), education interventions concerning EBP (intervention), without limiting comparison and outcomes, and randomized control trial (RCT) or non-randomized control trial (nRCT) designs. A literature review was conducted to gather raw data for the meta-analysis, focusing on publications from 1 January 2011 to 31 December 2023 to explore recent research trends and reflect the developments in EBP education for undergraduate nursing.

### 2.3. Data Collection and Selection Processes

First, we searched the Medical Subject Headings (MeSH) term in PubMed, EMTREE in the Excerpta Medica database (EMBASE), and Cumulative Index to Nursing and Allied Health (CINAHL) headings to identify the relevant keywords and index terms. The keywords were (1) “Students, Nursing” [MeSH], (2) “Evidence-Based Practice” [MeSH] OR “Evidence-Based Nursing” [MeSH], (3) “Education” [MeSH] OR “Learning” [MeSH]” and other index terms. Second, we used all the identified keywords and index terms to search for relevant articles by using adequately AND/OR. We selected that English language articles were searched for in Cochrane Library, PubMed, EMBASE, CINAHL, PsycINFO, and Web of Science. Additionally, Korean language articles were searched for using the Research Information Service System DBpia. Third, we adhered to the PRISMA guidelines. We initiated our search through various databases, followed by the removal of duplicate entries using the bibliographic management program (Covidence, Melbourne, Australia).

**Figure 1:**
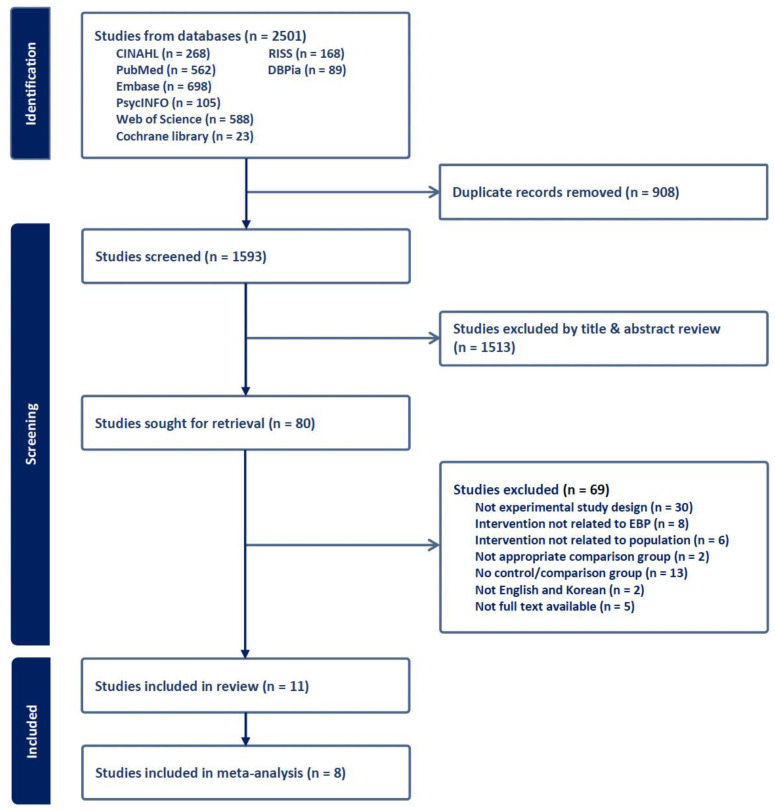
PRISMA diagram of the study screening.

The literature search and the titles and abstracts of the remaining articles were performed independently by two researchers. We then examined the full texts of these studies in detail to determine their suitability for inclusion in our review. In instances where opinions differed, a consensus was reached through discussion among three researchers. The literature selection process is illustrated in the PRISMA flow diagram (Figure 1). A detail search strategy is presented in Supplementary Table S2.

Initially, 2501 articles were retrieved—of which, 908 duplicates were removed. Subsequently, two researchers independently reviewed the titles and abstracts of the remaining 1593 articles. After applying the inclusion and exclusion criteria, 80 articles were selected for full-text review based on their eligibility. Ultimately, 11 articles were chosen for the systematic review, with 8 included in the meta-analysis (Figure 1).

### 2.4. Risk of Bias Assessment

To assess the quality of the selected articles, the Risk of Bias (RoB) II tool [16] was applied for the RCT studies, while the Risk of Bias In Non-randomized Studies—of Interventions (ROBINS-I) tool [17] was used for quasi-experimental nRCT studies. For RCTs, the RoB II tool evaluates five domains, including “bias arising from the randomization process”, “bias due to deviations from intended interventions”, “bias due to missing outcome data”, “bias in measurement of the outcome”, and “bias in selection of the reported result”. For each domain, risk of bias is evaluated as “Yes”, “Probably yes”, “Probably no”, “No”, or “No information”, and an evaluation algorithm ultimately categorizes the domain as showing “low risk”, “some concerns”, or “high risk”.

For nRCT studies, the ROBINS-I tool assesses “bias due to confounding”, “bias due to selection of participants”, “bias in classification of interventions”, “bias due to deviations from intended interventions”, “bias due to missing data”, “bias in the measurement of outcomes”, and “bias in the selection of reported results”. For each of these 7 domains, the risk of bias is evaluated as “Yes”, “Probably yes”, “Probably no”, “No”, or “No information” and is ultimately classified as “low”, “moderate”, “serious”, or “critical” by an evaluation algorithm.

The risk of bias assessment for the selected articles was performed by four researchers who independently rated the quality of each article. When the reviewers disagreed about an assessment, consensus was achieved through discussion.

### 2.5. Data Extraction

For the systematic literature review, the selected articles were documented with information on the authors, publication year, study participants’ school year, average age, design, intervention details, outcomes, and results using Microsoft Excel 2020. A total of 11 articles were subjected to the systematic review, and among these, the data available for meta-analysis from 8 articles included “EBP competency” (n = 7), “critical thinking” (n = 3), and “problem-solving ability” (n = 2).

### 2.6. Data Analysis

The meta-analysis was primarily conducted using R version 3.6.3. All outcomes analyzed were continuous variables. When studies used different measurement tools, we employed the standard mean difference (SMD) and 95% confidence intervals (CIs) as effect size indicators [18]. In our analysis, the weights of individual effect sizes were calculated using the inverse variance method. Given the diversity in research methodologies, samples, interventions, and assessment tools across the studies, we used a random effects model to estimate the average effect size. The heterogeneity of the effect sizes was evaluated using the proportion of observed variance (I²). We interpreted the degree of heterogeneity based on the I² value: I² of 25% or less indicated low heterogeneity, I² over 25% but under 75% indicated moderate heterogeneity, and an I² of 75% or more was considered high heterogeneity. When the heterogeneity was high in effect sizes, a meta-regression was conducted according to the moderating variables to explore possible reasons for the heterogeneity.

To assess the publication bias, we visually inspected the symmetry of a funnel plot. To verify asymmetry between the effect size and standard error, the Begg’s test was conducted. We interpreted that, if the results were statistically non-significant, publication bias did not significantly impact the interpretation of our meta-analysis results.

To assess the robustness of the synthesized results, we conducted the sensitivity analyses to obtain the impact of excluding certain data points on the overall effect size of our meta-analysis.

## 3. Results

### 3.1. Characteristics of the Studies Selected for Systematic Review

The characteristics of the 11 studies [19,20,21,22,23,24,25,26,27,28,29] are summarized in Table 1 and detailed further in Supplementary Table S3. Of the 11 articles, 9 studies were nRCTs and 2 were RCTs reported between 2015 and 2023. The studies were conducted across five countries: China (n = 1), Iran (n = 1), Portugal (n = 1), South Korea (n = 6), and Spain (n = 2), with participant numbers ranging from 21 to 152. The participants were primarily 2nd, 3rd, and 4th year nursing students, with the majority being 4th year students (n = 7). The educational format predominantly centered on face-to-face lectures supplemented by group discussions, team activities, and presentations. Other formats included web-based (n = 1), virtual education (n = 1), and flipped classroom (n = 1), along with a high-fidelity simulation practicum (n = 1) and clinical practicum (n = 1). The duration of the interventions varied, lasting from as little as 5 days (n = 2) to 4–7 weeks (n = 4) and up to 12–17 weeks (n = 5).

All the studies described EBP education interventions that covered some or all the EBP steps: (1) asking a question, (2) finding the evidence, (3) appraising the evidence, (4) implementing it into clinical practice, and (5) evaluating the evidence. One study specifically implemented the intervention using EBCPG in a fundamental nursing class.

The tools used to measure the variables for the meta-analysis included EBP competency was evaluated using the Evidence-Based Practice Evaluation Competence Questionnaire (EBP-COQ) by Ruzafa-Martinez et al. [30] (n = 3), the Essential Competencies for EBP in Nursing by Stevens [31] (n = 2), the Evidence-Based Practice Questionnaire (EBPQ) by Upton and Upton [32] (n = 1), and the Evidence-Based Medicine Competencies by Park et al. [33] (n = 1). Critical thinking was assessed using the Critical Thinking Disposition Scale for Nursing Students by Kwon et al. [34] (n = 3), and problem-solving ability was measured with a tool developed by Marshall [35] (n = 2).

**Table 1:** General characteristics of the included studies.

Ref No.	Author (Years) Country	Study Design	Participants (1) Grade (2) Total (*N*), Exp (n)/Cont (n)	Intervention		Variables (Effect)	Measurements (1) EBP Competency (2) Critical Thinking (3) Problem-Solving Ability
				Program (1) Exp (2) Cont	Duration (1) Weeks or Days (2) Hours (3) Sessions		
[19] ^†^	Jang (2015) South Korea	nRCT	(1) 2nd (2) 45, 22/23	(1) EBN Course using action learning (2) No intervention	(1) 7 weeks (2) 14 h (3) 7	EBN competency (+) EBN knowledge (+) EBN pursuit towards (+) EBN practice (NS) Information literacy (+) Proactivity in problem solving (+)	(1) Evidence-based medicine competencies [33] (2) Not measured (3) Team skills questionnaire [35]
[20] ^†^	Ruzafa-Martínez (2016) Spain	nRCT	(1) 2nd, 3rd (2) 120, 61/59	(1) EBP course (2) No intervention	(1) 15 weeks (2) 150 h (3) Not indicated	EBP competence (+) EBP attitude (+) EBP knowledge (+) EBP skills (+)	(1) EBP-COQ [30]
[21] ^†^	Kim (2018) South Korea	nRCT	(1) 2nd (2) 105, 52/53	(1) EBP in fundamental nursing class (2) Traditional class	(1) 8 weeks (2) 24 h (3) 4	Proactivity in problem solving (+) Future use of EBP (+) Critical thinking deposition (NS)	(1) Not measured (2) Korean critical thinking deposition [34] (3) Team skills questionnaire [35]
[22] ^†^	Kim (2019) South Korea	nRCT	(1) 4th (2) 44, 22/22	(1) EBP education program (2) No intervention	(1) 4 weeks (2) 20 h (3) 8	EBP knowledge (+) EBP skills (+) EBP attitudes (+) EBP competencies (+) Future use of EBP (+) Critical thinking (+)	(1) EC for EBP [31] (2) Not measured (3) Korean critical thinking deposition [34]
[23]	Oh (2019) South Korea	nRCT	(1) 4th (2) 45, 21/24	(1) EBP education program (2) No intervention	(1) 5 days (2) 30 h (3) 6	EBP knowledge (+) EBP self-efficacy (+) Resource utilization (+) Database utilization (+)	(1) Not measured (2) Not measured (3) Not measured
[24] ^†^	Lee (2020) South Korea	nRCT	(1) 4th (2) 48, 24/24	(1) Simulation practicum EBP education (2) Simulation practicum	(1) 6 weeks (2) 24 h (3) 6	EBP knowledge (+) EBP attitude (+) EBP competency (+) Future use of EBP (+) Critical thinking (+)	(1) EC for EBP [31] (2) Not measured (3) Korean critical thinking deposition [34]
[25] ^†^	Park (2020) South Korea	nRCT	(1) 4th (2) 81, 41/40	(1) Web-based EBP education (2) Traditional teaching	(1) 5 days (2) Not indicated (3) 6	EBP competence (+) EBP knowledge and skills (+) EBP attitude (NS) EBP practice (NS) Clinical-questioning confidence (+)	(1) EBPQ [32] (2) Not measured (3) Not measured
[26]	Cardoso (2021) Portugal	Cluster RCT	(1) 8th semester (2) 148, 74/74	(1) EBP education program (2) Traditional teaching	(1) 17 weeks (2) 18 h (3) 6	EBP knowledge and skills (+)	(1) Not measured (2) Not measured (3) Not measured
[27]	Shamsaee (2021) Iran	RCT	(1) 6th and 8th semester (2) 79, 39/40	(1) Virtual education on information literacy for EBP (2) No intervention	(1) 4 weeks (2) Not indicated (3) 6	Use of different information resources (NS) Information searching skills (+) Knowledge about search operators (+) Selecting the most appropriate search statement (+)	(1) Not measured (2) Not measured (3) Not measured
[28] ^†^	Du (2023) China	nRCT	(1) 3rd (2) 258, 126/132	(1) EBP in nursing research (2) Conventional teaching	(1) 12 weeks (2) 36 h (3) 9	EBP attitude (+) EBP skills (+) EBP knowledge (NS) EBP overall score (+) Learning satisfaction (NS) Performance of team’s research protocol (+)	(1) EBP-COQ [30] (2) Not measured (3) Not measured
[29] ^†^	Ruzafa-Martínez (2023) Spain	nRCT	(1) 4th (2) 295, 143/152	(1) EBP with flipped classroom (2) Traditional teaching	(1) 15 weeks (2) 150 h (3) 6	EBP attitude (+) EBP knowledge (NS) EBP skills (+) EBP competence (+) Final exam (NS)	(1) EBP-COQ [30] (2) Not measured (3) Not measured

Cont: Control group; EBN: Evidence-based nursing; EBP: Evidence-based practice; EBP-COQ: Evidence-based practice competency questionnaire; EBPQ: Evidence-based practice questionnaire; EC for EBP: Essential competencies for evidence-based practice; Exp: Experimental group; H: Hours; nRCT: non-Randomized controlled trial; NS: Not significant; RCT: Randomized controlled trial; Ref: Reference; ^†^: Meta-analysis included; (+): Significant changes.

### 3.2. Quality Assessment

**Figure 2:**
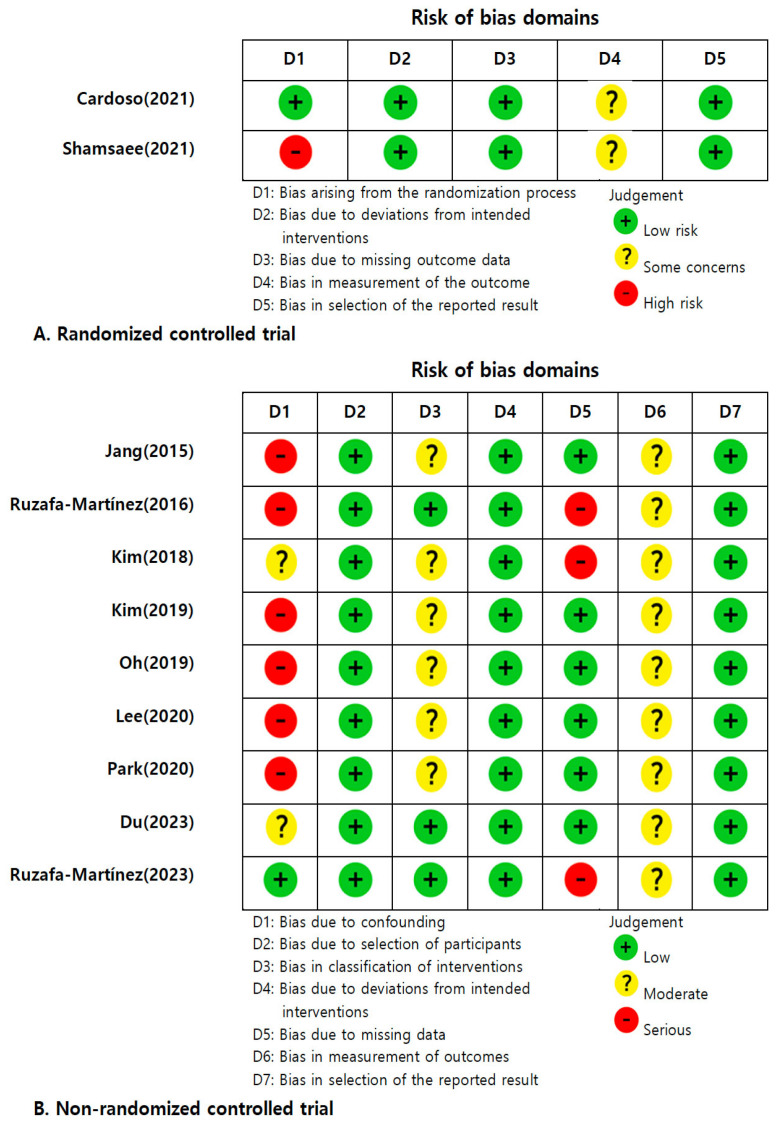
Quality assessment [19,20,21,22,23,24,25,26,27,28,29].

The quality assessment results for 11 studies are presented in Figure 2. For the risk of bias assessment, two RCTs were evaluated using the RoB II tool, and nine nRCTs were assessed with the ROBINS-I tool. In evaluating the two RCTs, one study (50%) was found to have a “high risk” in the randomization process. Among the nine nRCTs, six studies (66.7%) were judged to be “serious” with confounding factors, and three studies (33.3%) were “serious” for missing data. All the studies (100%) were assessed to have be “moderate” in the measurement of the outcomes. Overall, eight of the studies (72.7%) indicated at least one “serious” concern across all seven domains, and all the studies were included in the final analysis.

### 3.3. Effectiveness of EBP Education

#### 3.3.1. EBP Competency

**Figure 3:**
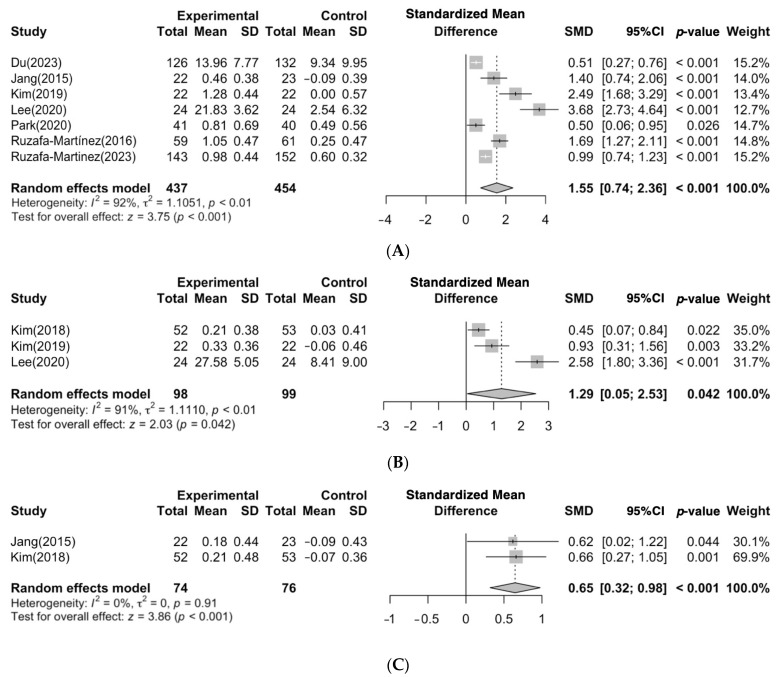
(**A**) Effect of EBP education programs on EBP competency. (**B**) Effect of EBP education programs on critical thinking. (**C**) Effect of EBP education programs on problem-solving ability. The x-axis represents the effect sizes measured as Standardized Mean Differences. The gray boxes in the figure indicate the effect size of individual studies, with the horizontal line inside each box representing the median effect size. The vertical lines extending from each box depict the 95% confidence intervals. The diamond-shaped figure centrally located below the zero line reflects the overall effect size calculated from the combined data of all studies, with its width representing the overall confidence interval. The white and black line in Figure 3A have the same meaning [19,20,21,22,24,25,28,29].

The overall effect size of seven studies reporting on the effectiveness of EBP competency was determined to be 1.55, with a statistically significant 95% CI ranging from 0.74 to 2.36 (Z = 3.75, *p* = < 0.001) (Figure 3A). These studies were reported a high degree of heterogeneity (I^2^ = 92%).

Further meta-regression analyses showed the impact of grade and duration as moderating variables (Table 2). The 4th grade group reported a higher effect size (1.84) compared to the 2nd and 3rd grade groups (1.20), with a significant difference in the effect sizes (*p* = 0.001, I^2^ = 95.8%). Analyzing with duration as the moderating variable, the effect size for the 4–7 weeks group had the highest effect size of 2.47, statistically significant, and the heterogeneity was high (*p* < 0.001, I^2^ = 94.4).

#### 3.3.2. Critical Thinking

The overall effect size of three studies reporting on the effectiveness of critical thinking was determined to be 1.29, with a statistically significant 95% CI ranging from 0.05 to 2.53 (Z = 2.03, *p* = 0.042) (Figure 3B). These studies reported a high degree of heterogeneity (I^2^ = 91%).

#### 3.3.3. Problem-Solving Ability

The overall effect size of two studies reporting on the effectiveness of problem-solving ability was determined to be 0.65, with a statistically significant 95% CI ranging from 0.32 to 0.98 (Z = 3.86, *p* = < 0.001) (Figure 3C).

### 3.4. Publication Bias

**Figure 4:**
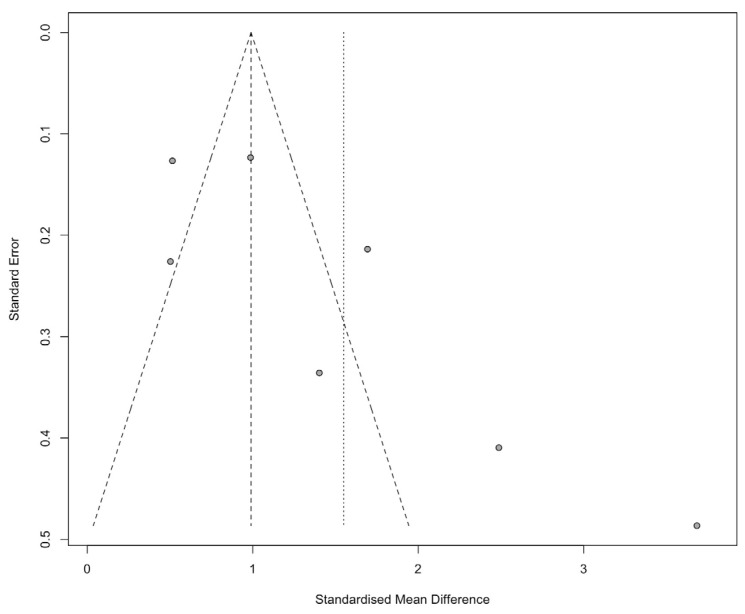
Funnel plot of the results. The x-axis shows the Standardized Mean Difference (SMD) for each study, while the y-axis represents the standard error, inversely related to study size. Circles represent individual studies, expected to form a symmetric funnel around the vertical line at SMD = 1. The dashed lines outline the expected distribution if no bias is present. Deviations from this symmetry may indicate bias.

The visual inspection of publication bias for the studies included in the meta-analysis showed that the funnel plot was not generally symmetrical, and the presence of some studies outside the triangle suggests a possible publication bias. However, the results from Begg’s test indicated that the publication bias was not significant (Kendall’s tau = 0.62, *p* = 0.069) (Figure 4).

Our sensitivity analysis demonstrated that excluding specific data points from our meta-analysis led to a lower effect size than the original 1.55. Therefore, we retained the original dataset to ensure the robustness of the synthesized results.

## 4. Discussion

This systematic review and meta-analysis synthesized findings from multiple studies to assess the impact of EBP education on undergraduate nursing students. Our findings indicate that EBP education effectively enhances EBP competency, critical thinking, and problem-solving abilities among undergraduate nursing students.

Firstly, EBP education significantly improved nursing students’ EBP competency, demonstrating a notable effect size of 1.55. EBP competency encompasses knowledge, skills, and behaviors [33]. Among seven studies included in the meta-analysis, five studies [19,20,25,28,29] measured EBP competency, including knowledge, attitudes, and skills for EBP, while two studies [22,24] assessed knowledge and skills as separate variables rather than subdomains of EBP competency.

Although it is challenging to make direct comparisons with previous meta-analyses of EBP education among nurses [14], Sapri et al. [14] analyzed EBP knowledge, skills, and attitudes among nurses, finding significant effect sizes of 0.48 for EBP knowledge/skills and 0.39 for attitudes toward EBP. They noted the effectiveness of nurses’ EBP knowledge/skills across a variety of educational strategies that included lectures, group discussions, clinical case scenarios, and a training duration of 16–24 h. Similarly, in our study, EBP education extended beyond lectures to include practicums that utilized clinical scenarios, group discussions, and activities, proving beneficial for addressing challenges in EBP implementation in clinical settings [22]. Additionally, the EBP education in our study comprehensively covered the five steps of EBP [1]. This multifaceted approach, integrating theory with practical application, likely contributed to the enhanced competency observed among our study participants.

The study identified high heterogeneity among the literature, leading to a meta-regression analysis. It revealed that EBP competency was significantly higher in settings involving 4th grade students and during interventions lasting 4–7 weeks. Specifically, the effect size for 4th grade students was 1.84, significantly higher than that for 2nd and 3rd grade students, which was 1.20. According to Kim et al. [22], as students progress through their education and gain clinical experience, they recognize the importance of EBP. Similarly, a meta-analysis on the impact of EBP education among nurses indicated that those with higher educational levels exhibited significantly greater EBP knowledge, skills, and behaviors [14]. Burke et al. [36] noted that EBP competency content varies by educational level—undergraduate through doctoral—and highlighted the importance of a grade-specific approach in undergraduate nursing programs. Consequently, implementing educational strategies that enhance EBP competency progressively based on the student’s year or level seems effective.

When analyzing with duration as a moderating variable, the effect size for 4–7 weeks was 2.47, which was statistically significantly the highest. Although not a direct comparison, a meta-analysis on the effect of EBP education among nurses [14] showed previous studies have shown that, the longer the intervention duration (8 weeks), the higher the effect size on EBP attitude and behavior. However, there was no difference in duration in EBP knowledge and skills [14]. Further research should explore the optimal duration for various components of EBP competency to ensure that interventions are tailored effectively.

Additionally, four measurement items—knowledge, attitudes, skills, and practice—were used to assess EBP competency in this study [30,31,32,33]. Despite observing high heterogeneity in the research, there is a clear need for future studies to consider standardizing measurement tools for EBP competency and exploring ways to ensure consistency among these measures.

EBP competency is an essential skill that nurses must master to become competent and professional in clinical settings, and its importance has been continuously emphasized [37]. Therefore, it is necessary to provide nursing students with opportunities to apply EBP knowledge gained from lectures and practicums directly in clinical settings, thus preparing them to make quick and effective decisions in complex clinical environments.

Secondly, EBP education has been demonstrated to significantly enhance critical thinking in nursing students, as shown by a robust effect size of 1.29. The three studies [21,22,24] included in our meta-analysis assessed critical thinking using a tool developed by Kwon et al. [34]. While a different measurement tool was employed, the findings from a meta-analysis by Cui et al. [38], which evaluated the efficacy of EBN education among higher vocational students, undergraduates, and postgraduates, aligned with our results concerning the tools used for critical thinking. In contrast, Kim et al. [21], whose study was also included in our analysis, reported no significant impact of EBP education on critical thinking. Kim et al. [21] suggested that critical thinking needs to be developed progressively through a sustained and systematic educational approach starting at the undergraduate level. Consequently, due to these mixed results, further research is needed, particularly to explore the effects of EBP education on critical thinking in undergraduate nursing students.

Thirdly, EBP education has proven effective in boosting the problem-solving abilities of nursing students, evidenced by an effect size of 0.65. EBP education, which includes formulating PICO questions and applying the five-step EBN process, enhances students’ abilities to correctly approach clinical problems and find accurate solutions [7]. Notably, both studies [19,21] included in our meta-analysis found that team activities significantly improved problem-solving skills. Such activities allow students to integrate diverse perspectives and experiences, helping them navigate analytical processes to develop innovative solutions to complex clinical challenges [39]. However, since only two studies [19,21] examined the effect of EBP education on problem-solving abilities, additional research is needed to validate these findings and more thoroughly assess the impact.

The varied influence of EBP education on competency, critical thinking, and problem-solving ability was evident. A meta-regression analysis indicated that educational strategies, particularly those tailored for specific student levels and lasting between 4 and 7 weeks, enhance the effectiveness of EBP education. This highlights the need for flexible EBP curricula that cater to the diverse learning needs of nursing students, highlighting the importance of developing curricula that can adapt to various student levels. Continuous research is essential to deepen our understanding of the significance of EBP education in clinical settings and to optimize its implementation. Such studies will enhance the clinical relevance of EBP in nursing education, ultimately preparing nurses to make effective decisions in clinical situations.

The novelty of this study lies in its meta-analysis of existing EBP education programs, which assesses how they enhance competencies, critical thinking, and problem-solving in undergraduate nursing students. Moreover, it is meaningful to analyze the impact of different educational durations and grade levels on EBP competency among these students using meta-regression.

## 5. Conclusions

This study has demonstrated that EBP education positively impacts undergraduate nursing students’ EBP competency, critical thinking, and problem-solving ability. The meta-regression analysis suggested that specific educational settings, such as those tailored for 4th grade students or structured within duration limits of 4–7 weeks, can enhance the efficacy of EBP education. These findings support the development of a customizable and applied EBP education actively for students, preparing nursing students to effectively implement EBP in clinical settings after graduation. However, due to the high heterogeneity, cautious interpretation is necessary.

## 6. Limitations

This study is subject to several limitations. Firstly, the systematic review was constrained to studies published in Korean and English within a predefined period. This selection criterion potentially excludes relevant unpublished studies or those published in other languages, thereby limiting the comprehensiveness of the review. Secondly, the inherent diversity in sample sizes, content, and methodologies among the studies included in the meta-analysis might have biased the results. Thirdly, basing the meta-analysis on only eight studies may lead to the overestimation or underestimation of effect sizes. Therefore, cautious interpretation should be considered. The findings are tentative, given the high heterogeneity observed, underscoring the necessity for more refined studies focusing on variables such as grade, duration, and measurement. Finally, while Begg’s test indicated no significant publication bias, the asymmetry observed in the funnel plot and the presence of some studies outside the expected triangle raise concerns of potential bias. These observations may be particularly impactful due to the relatively small number of studies included in the meta-analysis, which can limit the power of statistical tests to detect bias. This underscores the need for caution in interpreting the results and suggests that future research should extend to a meta-analysis of more comprehensive EBP educational interventions and consider additional variables not accounted for in this study.

## Figures and Tables

**Table 2:** Effect of the moderator variables.

Variables	Categories	k	ES	SE	Z	*p*	95% CI		I^2^	*p*
							Lower Limit	Upper Limit		
Grade	2nd, 3rd	3	1.20	0.66	1.81	0.071	−0.10	2.49	95.8	0.001
	4th	4	1.84	0.58	3.16	0.002	0.70	2.99		
Duration	<1 week	1	1.06	0.47	2.23	0.026	0.13	1.98	94.4	<0.001
	4–7 weeks	3	2.47	0.52	4.75	0.548	1.45	3.49		
	12–15 weeks	3	0.50	0.84	0.60	<0.001	−1.14	2.14		

CI = Confidence interval; ES = Effect size; SE = Standard error.

## Data Availability

All studies in our study are included in the Supplementary Materials.

## Supplementary Materials

The following supporting information can be downloaded at https://www.mdpi.com/article/10.3390/ijerph21050637/s1: Table S1: PRISMA Checklist; Table S2: Search Strategy; Table S3: Summary of selected studies for systematic review and meta-analysis.

## References

[1] Melnyk B.M., Fineout-Overholt E. (2022). Evidence-Based Practice in Nursing & Healthcare: A Guide to Best Practice.

[2] International Council of Nurses (2018). Position Statements: Evidence Based Nursing Staffing. https://www.icn.ch/nursing-policy/position-statements.

[3] Albarqouni L., Hoffmann T., Straus S., Olsen N.R., Young T., Ilic D., Shaneyfelt T., Haynes R.B., Guyatt G., Glasziou P. (2018). Core competencies in evidence-based practice for health professionals: Consensus statement based on a systematic review and delphi survey. JAMA Netw. Open.

[4] Li S., Cao M., Zhu X. (2019). Evidence-based practice: Knowledge, attitudes, implementation, facilitators, and barriers among community nurses-systematic review. Medicine.

[5] Horntvedt M.-E.T., Nordsteien A., Fermann T., Severinsson E. (2018). Strategies for teaching evidence-based practice in nursing education: A thematic literature review. BMC Med. Educ..

[6] Melynk B.M., Gallagher-Ford L., Long L.E., Fineout-Overholt E. (2014). The establishment of evidence-based practice competencies for practicing registered nurses advanced practice nurses in real-world clinical settings: Proficiencies to improve healthcare quality, reliability, patient outcomes, and costs. Worldviews Evid.-Based Nurs..

[7] Ilic D. (2009). Assessing competency in evidence based practice: Strengths and limitations of current tools in practice. BMC Med. Educ..

[8] Papathanasiou V.I., Kleisiaris F.C., Fradelos E.C., Kakou K. (2014). Critical thinking: The development of an essential skill for nursing students. Acta Inform. Med..

[9] Majid S., Foo S., Luyt B., Zhang X., Chang Y.K., Mokhtar I.A. (2011). Adopting evidence-based practice in clinical decision making: Nurses’ perceptions, knowledge, and barriers. J. Med. Libr. Assoc..

[10] Keiffer M.R. (2018). Engaging nursing students: Integrating evidence-based inquiry, informatics, and clinical practice. Nurs. Educ. Perspect..

[11] Patelarou A.E., Mechili E.A., Ruzafa-Martinez M., Dolezel J., Gotlib J., Skela-Savič B., Ramos-Morcillo A.J., Finotto S., Jarosova D., Smodiš M. (2020). Educational interventions for teaching evidence-based practice to undergraduate nursing students: A scoping review. Int. J. Environ. Res. Public Health.

[12] Albarqouni L., Hoffmann T., Glasziou P. (2018). Evidence-based practice educational intervention studies: A systematic review of what is taught and how it is measured. BMC Med. Educ..

[13] Howard B., Diug B., Ilic D. (2022). Methods of teaching evidence-based practice: A systematic review. BMC Med. Educ..

[14] Sapri N.D., Ng Y.T., Wu V.X., Klanin-Yobas P. (2022). Effectiveness of educational interventions on evidence-based practice for nurses in clinical settings: A systematic review and meta-analysis. Nurse Educ. Today.

[15] Moher D., Shamseer L., Clarke M., Ghersi D., Liberati A., Petticrew M., Shekelle P., Stewart L.A., Prisma-P Group (2015). Perferred reporting items for systematic review and meta-analysis protocols (PRISMA-P) 2015 statement. Syst. Rev..

[16] Cochrane Methods Bias RoB 2: A Revised Cochrane Risk-of-Bias Tool for Randomized Trials. https://methods.cochrane.org/bias/resources/rob-2-revised-cochrane-risk-bias-tool-randomized-trials.

[17] Kim S.Y., Park J.E., Seo H.J., Shin S.S., Lee S.J., Jang B.H., Cha Y.J., Choi M.Y., Park J.Y., Suh Y.S. (2021). NECA’s Guidance for Assessing Tools of Risk of Bias.

[18] Morris S.B., DeShon R.P. (2002). Combining effect size estimates in meta-analysis with repeated measures and independent-groups designs. Psychol. Methods.

[19] Jang K.S., Kim E.A., Park H. (2015). The effects of an evidence-based nursing course using action learning on undergraduate nursing students. J. Korean Acad. Soc. Nurs. Educ..

[20] Ruzafa-Martínez M., López-Iborra L., Barranco D.A., Ramos-Morcillo A.J. (2016). Effectiveness of an evidence-based practice (EBP) course on the EBP competence of undergraduate nursing students: A quasi-experimental study. Nurse Educ. Today.

[21] Kim Y.-H., Ryu S., Kim H. (2018). Effect of fundamental nursing class using evidence-based practice. J. Korea Contents Assoc..

[22] Kim J.S., Gu M.O., Chang H. (2019). Effects of an evidence-based practice education program using multifaceted interventions: A quasi-experimental study with undergraduate nursing students. BMC Med. Educ..

[23] Oh E.G., Yang Y.L. (2019). Evidence-based nursing education for undergraduate students: A preliminary experimental study. Nurse Educ. Pract..

[24] Lee S., Cho K. (2020). Effects of simulation practicum using evidence-based practice practical education program. J. Korean Assn. Lean. Cent. Curric. Instr..

[25] Park M., Jeong M., Lee M., Cullen L. (2020). Web-based experiential learning strategies to enhance the evidence-based-practice competence of undergraduate nursing students. Nurse Educ. Today.

[26] Cardoso D., Couto F., Cardoso A.F., Bobrowicz-Campos E., Santos L., Rodrigues R., Coutinho V., Pinto D., Ramis M.A., Rodrigues M.A. (2021). The effectiveness of an evidence-based practice educational program on undergraduate nursing students’ EBP knowledge and skills: A cluster randomized control trial. Int. J. Environ. Res. Public Health.

[27] Shamsaee M., Mangolian Shahrbabaki P., Ahmadian L., Farokhzadian J., Fatehi F. (2021). Assessing the effect of virtual education on information literacy competency for evidence-based practice among the undergraduate nursing students. BMC Med. Inform. Decis. Mak..

[28] Du S., Jin S., Zhang H., Chen L., Zhang Y. (2023). Incorporating evidence-based practice education in nursing research curriculum of undergraduate nursing students: A quasi-experimental study. Nurse Educ. Pract..

[29] Ruzafa-Martínez M., Molina-Rodríguez A., Pérez-Muñoz V., Leal-Costa C., Ramos-Morcillo A.J. (2023). Effectiveness of the flipped classroom methodology on the learning of evidence-based practice of nursing students: Quasi-experimental design. Nurse Educ. Today.

[30] Ruzafa-Martinez M., Lopez-Iborra L., Moreno-Casbas T., Madrigal-Torres M. (2013). Development and validation of the competence in evidence based practice questionnaire (EBP-COQ) among nursing students. BMC Med. Educ..

[31] Stevens K.R. (2009). Essential Competencies for Evidence-Based Practice in Nursing.

[32] Upton D., Upton P. (2006). Development of an evidence-based practice questionnaire for nurses. J. Adv. Nurs..

[33] Park J., Yoon T., Ko J. (2009). Development and validation of a questionnaire to evaluate medical students’ evidence-based medicine competencies. Korean J. Med. Educ..

[34] Kwon I.S., Lee G.E., Kim G.D., Kim Y.H., Park K.M., Park H.S., Sohn S.K., Lee W.S., Jang K.S., Chung B.Y. (2006). Development of a critical thinking disposition scale for nursing students. J. Korean Acad. Nurs..

[35] Marshall L.C. (2003). The Relationship between Efficacy, Teamwork, Effort and Patient Satisfaction. Ph.D. Thesis.

[36] Burke L.E., Schlenk E.A., Sereika S.M., Cohen S.M., Happ M.B., Dorman J.S. (2005). Developing research competence to support evidence-based practice. J. Prof. Nurs..

[37] Reid J., Briggs J., Carlisle S., Scott D., Lewis C. (2017). Enhancing utility and understanding of evidence based practice through undergraduate nurse education. BMC Nurs..

[38] Cui C., Li Y., Geng D., Zhang H., Jin C. (2018). The effectiveness of evidence-based nursing on development of nursing students’ critical thinking: A meta-analysis. Nurse Educ. Today.

[39] Aglen B. (2016). Pedagogical strategies to teach bachelor students evidence-based practice: A systematic review. Nurse Educ. Today.

