# Supplementary material for: Effectiveness of an Evidence-Based Practice Education Program for Undergraduate Nursing Students: A Systematic Review and Meta-Analysis
Source: Int J Environ Res Public Health. 2024 May 16;21(5):637. doi: 10.3390/ijerph21050637 (PMC11121330; doi:10.3390/ijerph21050637)
Supplement: Supplementary file 1 [file ijerph-21-00637-s001.zip › ijerph-2953867-supplementary.pdf]

**Supplementary Table S1:** PRISMA Checklist

| Section and Topic | Item # | Checklist item | Location where item is reported |
|---|---|---|---|
| **TITLE** | | | |
| Title | 1 | Identify the report as a systematic review. | p.1 |
| **ABSTRACT** | | | |
| Abstract | 2 | See the PRISMA 2020 for Abstracts checklist. | p.1 |
| **INTRODUCTION** | | | |
| Rationale | 3 | Describe the rationale for the review in the context of existing knowledge. | p.1-2 |
| Objectives | 4 | Provide an explicit statement of the objective(s) or question(s) the review addresses. | p.2 |
| **METHODS** | | | |
| Eligibility criteria | 5 | Specify the inclusion and exclusion criteria for the review and how studies were grouped for the syntheses. | p.2 |
| Information sources | 6 | Specify all databases, registers, websites, organisations, reference lists and other sources searched or consulted to identify studies. Specify the date when each source was last searched or consulted. | p.3 |
| Search strategy | 7 | Present the full search strategies for all databases, registers and websites, including any filters and limits used. | p.3, Supplmentary 2 |
| Selection process | 8 | Specify the methods used to decide whether a study met the inclusion criteria of the review, including how many reviewers screened each record and each report retrieved, whether they worked independently, and if applicable, details of automation tools used in the process. | p.3 |
| Data collection process | 9 | Specify the methods used to collect data from reports, including how many reviewers collected data from each report, whether they worked independently, any processes for obtaining or confirming data from study investigators, and if applicable, details of automation tools used in the process. | p.3 |
| Data items | 10a | List and define all outcomes for which data were sought. Specify whether all results that were compatible with each outcome domain in each study were sought (e.g. for all measures, time points, analyses), and if not, the methods used to decide which results to collect. | p.3 |
| | 10b | List and define all other variables for which data were sought (e.g. participant and intervention characteristics, funding sources). Describe any assumptions made about any missing or unclear information. | p.3 |
| Study risk of bias assessment | 11 | Specify the methods used to assess risk of bias in the included studies, including details of the tool(s) used, how many reviewers assessed each study and whether they worked independently, and if applicable, details of automation tools used in the process. | p.3 |
| Effect measures | 12 | Specify for each outcome the effect measure(s) (e.g. risk ratio, mean difference) used in the synthesis or presentation of results. | p.4 |
| Synthesis methods | 13a | Describe the processes used to decide which studies were eligible for each synthesis (e.g. tabulating the study intervention characteristics and comparing against the planned groups for each synthesis (item #5)). | p.4 |
| | 13b | Describe any methods required to prepare the data for presentation or synthesis, such as handling of missing summary statistics, or data conversions. | p.5 |
| | 13c | Describe any methods used to tabulate or visually display results of individual studies and syntheses. | p.5 |
| | 13d | Describe any methods used to synthesize results and provide a rationale for the choice(s). If meta-analysis was performed, describe the model(s), method(s) to identify the presence and extent of statistical heterogeneity, and software package(s) used. | p.5 |
| | 13e | Describe any methods used to explore possible causes of heterogeneity among study results (e.g. subgroup analysis, meta-regression). | p.5 |
| | 13f | Describe any sensitivity analyses conducted to assess robustness of the synthesized results. | p.5 |
| Reporting bias assessment | 14 | Describe any methods used to assess risk of bias due to missing results in a synthesis (arising from reporting biases). | p.4 |

| Section and Topic | Item # | Checklist item | Location where item is reported |
|---|---|---|---|
| Certainty assessment | 15 | Describe any methods used to assess certainty (or confidence) in the body of evidence for an outcome. | p.5 |
| **RESULTS** | | | |
| Study selection | 16a | Describe the results of the search and selection process, from the number of records identified in the search to the number of studies included in the review, ideally using a flow diagram. | p.5, Figure1 |
| | 16b | Cite studies that might appear to meet the inclusion criteria, but which were excluded, and explain why they were excluded. | Figure1 |
| Study characteristics | 17 | Cite each included study and present its characteristics. | p.5, Table 1, Supplementary 3 |
| Risk of bias in studies | 18 | Present assessments of risk of bias for each included study. | p.7, Figure2 |
| Results of individual studies | 19 | For all outcomes, present, for each study: (a) summary statistics for each group (where appropriate) and (b) an effect estimate and its precision (e.g. confidence/credible interval), ideally using structured tables or plots. | p.8-9 |
| Results of syntheses | 20a | For each synthesis, briefly summarise the characteristics and risk of bias among contributing studies. | p.8-9 Table 2 Figure 3A, 3B, 3C, 3D |
| | 20b | Present results of all statistical syntheses conducted. If meta-analysis was done, present for each the summary estimate and its precision (e.g. confidence/credible interval) and measures of statistical heterogeneity. If comparing groups, describe the direction of the effect. | |
| | 20c | Present results of all investigations of possible causes of heterogeneity among study results. | |
| | 20d | Present results of all sensitivity analyses conducted to assess the robustness of the synthesized results. | |
| Reporting biases | 21 | Present assessments of risk of bias due to missing results (arising from reporting biases) for each synthesis assessed. | p.7 |
| Certainty of evidence | 22 | Present assessments of certainty (or confidence) in the body of evidence for each outcome assessed. | p.10 |
| **DISCUSSION** | | | |
| Discussion | 23a | Provide a general interpretation of the results in the context of other evidence. | p.10-11 |
| | 23b | Discuss any limitations of the evidence included in the review. | p.11 |
| | 23c | Discuss any limitations of the review processes used. | p.11 |
| | 23d | Discuss implications of the results for practice, policy, and future research. | p.11 |
| **OTHER INFORMATION** | | | |
| Registration and protocol | 24a | Provide registration information for the review, including register name and registration number, or state that the review was not registered. | p.2 |
| | 24b | Indicate where the review protocol can be accessed, or state that a protocol was not prepared. | |
| | 24c | Describe and explain any amendments to information provided at registration or in the protocol. | |
| Support | 25 | Describe sources of financial or non-financial support for the review, and the role of the funders or sponsors in the review. | p.12 |
| Competing interests | 26 | Declare any competing interests of review authors. | p.12 |
| Availability of data, code and other materials | 27 | Report which of the following are publicly available and where they can be found: template data collection forms; data extracted from included studies; data used for all analyses; analytic code; any other materials used in the review. | p.12 |

**Supplementary Table S2:** Search Strategy

1) Pubmed and Cochrane library

#1 "Students, Nursing"[Mesh] OR "Education, Nursing, Baccalaureate"[Mesh]

#2 "Baccalaureate"[Title/Abstract] OR college[Title/Abstract] OR universit*[Title/Abstract] OR bachelor*[Title/Abstract] OR undergraduate[Title/Abstract]

#3 nurs*[Title/Abstract]

#4 student*[Title/Abstract]

#5 #1 OR (#2 AND #3 AND #4)

#6 "Evidence-Based Practice"[Mesh:NoExp] OR "Evidence-Based Nursing"[Mesh] OR "Evidence-based practice"[Title/Abstract] OR "Evidence-based nursing"[Title/Abstract]

#7 "Education, Nursing"[Mesh:NoExp] OR "Education"[Mesh:NoExp] OR "Learning"[Mesh:NoExp] OR. "Curriculum"[Mesh:NoExp] OR "Teaching"[Mesh:NoExp]

#8 "Lecture*"[Title/Abstract] OR "Program*"[Title/Abstract] OR "educat*"[Title/Abstract] OR "Class"[Title/Abstract] OR "Classes"[Title/Abstract] OR "Course*"[Title/Abstract] OR "Session*"[Title/Abstract] OR Intervention*[Title/Abstract] OR "Curricul*"[Title/Abstract]

#9 #7 OR #8

#10 #5 AND #6 AND #9

2) CINAHL

#1 (MH "Students, Nursing, Baccalaureate+") OR (MH "Students, Nursing+")

#2 TI ( undergraduate OR baccalaureate OR College OR Universit* OR bachelor*) OR AB ( undergraduate OR baccalaureate OR College OR Universit* OR bachelor*)

#3 (MH "Nurses+") OR TI(nurs*) OR AB(nurs*)

#4 (MH "Student+") OR TI (student*) OR AB (student*)

#5 #1 OR (#2 AND #3 AND #4)

#6 (MH "Nursing Practice, Evidence-Based+") OR (MH "Professional Practice, Evidence-Based+") OR TI( "evidence-based practice" OR "evidence-based nursing" ) OR AB( "evidence-based practice" OR "evidence-based nursing" )

#7 (MH "Education, Nursing+") OR (MH "Education, Nursing, Baccalaureate+") OR (MH "Lecture+") OR (MH "Curriculum+") OR (MH "Teaching+") OR (MH "Nursing interventions+")

#8 ( TI ( educat* OR program OR class* OR course* OR session* OR intervention* OR instruct* ) ) OR ( AB. ( educat* OR program OR class OR course* OR session* OR intervention* OR instruct* ) )

#9 #7 OR #8

#10 #5 AND #6 AND #9

3) PsycINFO

(((MAINSUBJECT.EXACT("Nursing") OR MAINSUBJECT.EXACT("Nurses") OR title("nurs*) OR abstract(" nurs*)) AND (MAINSUBJECT.EXACT("Students") OR title("student*) OR abstract("student*)) AND (title(undergraduate OR baccalaureate OR College OR Universit* OR bachelor*) OR abstract(undergraduate OR

baccalaureate OR College OR Universit* OR bachelor*))) OR MAINSUBJECT.EXACT("Nursing Students"))
AND
((MAINSUBJECT.EXACT("Evidence-based practice") OR MAINSUBJECT.EXACT("Evidence-based nursing") OR abstract("Evidence-based nursing") OR title("Evidence-based nursing") OR abstract("Evidence-based practice") OR title("Evidence-based practice"))
AND
(Title(educati* OR class OR classes OR course* OR session* OR invervention* OR instruct*) OR (MAINSUBJECT.EXACT("Education") OR MAINSUBJECT.EXACT("Learning") OR MAINSUBJECT.EXACT("Curriculum") OR MAINSUBJECT.EXACT("Teaching") OR MAINSUBJECT.EXACT("Training") OR MAINSUBJECT.EXACT("Programs") MAINSUBJECT.EXACT("Intervention")) OR Abstract(educati* OR class OR classes OR course* OR session* OR invervention* OR instruct*)))

4) EMBASE
#1 'baccalaureate nursing student'/exp
#2 undergraduate:ab,ti OR baccalaureate:ab,ti OR college:ab,ti OR universiti*:ab,ti OR bachelor*:ab,ti
#3 nurs*:ab,ti OR 'nurse'/exp
#4 student*:ab,ti OR 'student'/exp
#5 #1 OR (#2 AND #3 AND #4)
#6 ‘evidence based practice'/exp OR 'evidence based nursing'/exp OR 'evidence based practice':ab,ti OR 'evidence based nursing':ab,ti
#7 'undergraduate education'/exp OR 'learning'/exp OR 'teaching'/exp OR 'training'/exp OR 'intervention study'/exp OR 'program'/exp OR 'curriculum'/exp
#8 ‘educati*:ab,ti OR class:ab,ti OR course*:ab,ti OR session:ab,ti OR intervention*:ab,ti OR instruct*:ab,ti
#9 #7 OR #8
#10 #5 AND #6 AND #9

5) Web of Science
#1 baccalaureate OR College OR Universit* OR bachelor* OR undergraduate (Title) OR baccalaureate OR College OR Universit* OR bachelor* OR undergraduate (Abstract)
#2 nurs* (Title) OR nurs* (Abstract)
#3 student* (Title) OR student* (Abstract)
#4 #1 AND #2 AND #3
#5 evidence-based practice (Title) OR evidence-based practice (Abstract) OR evidence-based nursing (Title) OR evidence-based nursing (Abstract)
#6 class* OR course* OR curriculum* OR educati* OR intervention* OR program OR session* OR. teaching OR lecture (Title) OR class* OR course* OR curriculum* OR educati* OR intervention* OR program OR session* OR teaching OR lecture (Abstract)
#7 #4 AND #5 AND #6 "

**Supplementary Table S3:** Summary of selected studies for systematic review and meta-analysis.

| Ref No. | Author (years) Country | Study design | Participants 1)Grade 2)Total (*N*), Exp (n)/ Cont (n) | Age (Mean±SD) | Intervention: Program | Intervention: Methods | Intervention: Duration 1)Wks or days 2)Hours 3)Sessions | Control group | Attrition(%)/ITT/ Funding | Variables (effect) | Measurements 1)EBP competency 2)Critical thinking 3)Problem-solving ability |
|---|---|---|---|---|---|---|---|---|---|---|---|
| 19† | Jang (2015) South Korea | nRCT | 1)2nd<br>2)45, 22/23 | E:19.27±0.77<br>C:20.13±2.07 | EBN Course using action learning<br>-Overview EBN<br>-5 steps EBN<br>-Asking clinical questions<br>-Evidence research<br>-Critical appraisal<br>-Field work<br>-Final presentation | -Lecture<br>-Team activities<br>-Hands-on practice<br>-Field work<br>-Tutorial | 1) 7 wks<br>2) 14 hrs<br>3) 7 | No intervention | E:25->22(12%)<br>C:25->23(8%)<br>/No/No | -EBN Competency(p<.001)<br>-EBN knowledge(p<.001)<br>-EBN pursuit towards(p<.001)<br>-EBN practice(p=.176)<br>-Information literacy(p=.001)<br>-Proactivity in problem solving(p=.026) | 1)Evidence-based medicine competencies [33]<br>3)Team skills questionnaire [35] |
| 20† | Ruzafa-Martínez (2016) Spain | nRCT | 1)2nd ,3rd<br>2)120, 61/59 | E:20.39±2.85<br>C:22.52±6.51 | EBP course<br>-Introduction to EBP<br>-Clinical question formulation using the PICO format<br>-Designing and conducting the search for evidence in multiple evidence databases<br>-Evaluating and applying the evidence<br>-Final exercise in a clinical scenario | -Theoretical class<br>-Seminar | 1) 15 wks<br>2) 150 hrs (60h in class, 90h student works) | No intervention | E:75->59(21.3%)<br>C:73->61(16.4%)<br>/No/Yes | -EBP competence(p<.001)<br>-EBP attitude(p<.001)<br>-EBP knowledge(p<.001)<br>-EBP skills(p<.001) | 1)EBP-COQ [30] |
| 21† | Kim (2018) South Korea | nRCT | 1)2nd<br>2)105, 52/53 | E:21.30±0.97<br>C:21.90±1.82 | EBP in fundamental nursing class<br>-Using EBCPG about 'intravenous infusion', 'transfusion', 'enteral feeding', and 'indwelling urinary catheterization.'<br>-Making PICO | -Lecture<br>-Discussion<br>-Presentation | 1) 8 wks<br>2) 24 hrs<br>3) 4 | Traditional class | E:62->52(16.1%)<br>C:62->53(14.5%)<br>/No/Yes | -Proactivity in problem solving(p=.007)<br>-Future use of EBP(p=.012)<br>-Critical thinking deposition(p=.217) | 2)Korean critical thinking deposition [34]<br>3)Team skills questionnaire [35] |
| 22† | Kim (2019) South Korea | nRCT | 1)4th<br>2)44, 22/22 | E:22.82±0.40<br>C:23.18±0.52 | EBP education program using multifaceted interventions<br>-Introduction of EBP<br>-Clinical question<br>-Evidence search<br>-Critical appraisal<br>-Implementation and evaluation<br>-Integrative application of 5-step EBP | -Lecture<br>-Group discussion<br>-Presentation<br>-Computer lab | 1) 4 wks<br>2) 20 hrs<br>3) 8 | No intervention | E:23->22(4.3%)<br>C:24->22(8.3%)<br>/No/No | -EBP knowledge(p<.001)<br>-EBP skills(p<.001)<br>-EBP attitudes(p<.001)<br>-EBP competencies(p<.001)<br>-Future use of EBP(p=.001)<br>-Critical thinking(p<.001) | 1)EC for EBP [31]<br>3)Korean critical thinking deposition [34] |
| 23 | Oh (2019) South Korea | nRCT | 1)4th<br>2)45, 21/24 | E:23.52±0.93<br>C:23.70±1.04 | EBP education program<br>-Introduction of EBP, steps of EBP, defining clinical questions<br>-Finding evidence: literature searching strategies<br>-Critical appraisal of the evidence: study design and level of evidence<br>-Translating evidence into practice, evaluating outcomes of EBP, understanding of nursing sensitive outcomes | -Lectures (include special lectures from school librarian and clinical expert)<br>-TBL with flipped learning<br>-CBL<br>-PBL<br>-Group activity with student | 1) 5 days<br>2) 30 hrs<br>3) 6 | No intervention | E:22->21(4.5%)<br>C:26->24(7.7%)<br>/No/Yes | -EBP knowledge(p<.001)<br>-EBP self-efficacy(p<.001)<br>-Resource utilization(p=.009)<br>-Database utilization(p=.006) | |

| | | | | | | | | | | | |
|---|---|---|---|---|---|---|---|---|---|---|---|
| | | | | | -EBP actions in hospital, barriers and facilitators of EBP | presentation | | | | | |
| 24† | Lee<br>(2020)<br>South Korea | nRCT | 1)4th<br>2)48,<br>24/24 | E: 23.54±0.93<br>C:<br>23.42±0.78 | Simulation practicum EBP education<br>-Asking clinical questions<br>-Evidence research<br>-Critical appraisal<br>-Implementation<br>-Application of EBP | -Lecture<br>-Group discussion<br>-Presentation<br>-Practicum<br>-High fidelity simulator practicum | 1) 6 wks<br>2) 24 hrs<br>3) 6 | Simulation practicum | E:24->24(0%)<br>C:24->24(0%)<br>/No/No | -EBP knowledge(p<.001)<br>-EBP attitude(p<.001)<br>-EBP competency(p<.001)<br>-Future use of EBP(p<.001)<br>-Critical thinking(p<.001) | 1)EC for EBP [31]<br>3)Korean critical thinking deposition [34] |
| 25† | Park<br>(2020)<br>South Korea | nRCT | 1)4th<br>2)81,<br>41/40 | E:23.12±2.38<br>C:23.35±3.19 | Web-based EBP education<br>-EBP overview<br>-Clinical-question formulation: PICO<br>-Evidence search<br>-Critical appraisal<br>-Implementation<br>-Evaluation | -Animation based teaching<br>-Interactive game<br>-Interactive discussion<br>-Clinical practicum | 1) 5 days<br>2) Not indicated<br>3) 6 | Traditional teaching | E:44->41(6.8%)<br>C:42->40(4.8%)<br>/No/Yes | -EBP competence(p=.020)<br>-EBP knowledge and skills (p=.012)<br>-EBP attitude(p=.577)<br>-EBP practice(p=.250)<br>-Clinical-questioning confidence(p=.005) | 1)EBPQ [32] |
| 26 | Cardoso<br>(2021)<br>Portugal | Cluster RCT | 1)8th semester<br>2)148,<br>74/74 | E:22.20±2.84<br>C:21.70±1.42 | EBP education program<br>-Thinking about EBP<br>-Systematic review types<br>-Review question development<br>-Searching for studies<br>-Study selection process<br>-Data extraction<br>-Data synthesis | -Expository methods with practice tasks to groups<br>-Active methods through mentoring to groups | 1) 17 wks<br>2) 18 hrs<br>3) 6 | Education as usual | E:74->72(2.7%)<br>C:74->68(8.1%)<br>/Yes/Yes | -EBP knowledge and skills(p<.001) | |
| 27 | Shamsaee<br>(2021)<br>Iran | RCT | 1)6th and 8th semester<br>2)79,<br>39/40 | Not indicated | Virtual education on information literacy for EBP<br>-Perceiving the value of EBP in nursing<br>-Learning the level of skills required for undertaking different EBP activities<br>-Using example according to the steps of EBP<br>-Searching for studies<br>-Doing practical exercise<br>-Assessing and evaluating the studies | -Practice exercises<br>-Discuss | 1) 4 wks<br>2) Not indicated<br>3) 6 | No intervention | E:40->39(2.5%)<br>C:40->40(0%)<br>/No/No | -Use of different information resources(p=.18)<br>-Information searching skills(p=.002)<br>-Knowledge about search operators(p=.001)<br>-Selecting the most appropriate search statement(p=0.13) | |
| 28† | Du<br>(2023)<br>China | nRCT | 1)3rd<br>2)258,<br>126/132 | E:20.37±0.74<br>C:20.26±0.73 | EBP in nursing research<br>-Course orientation<br>-Developing and refining a research question<br>-Review of literature search<br>-Core elements of research design<br>-Principles of research design<br>-Types of nursing research design<br>-Sampling<br>-Data collection methods<br>-Academic paper writing in nursing and healthcare | | 1) 12 wks<br>2) 36 hrs<br>3) 9 | Conventional teaching | E:127->126(0.8%)<br>C:132->132(0%)<br>/Yes/Yes | -EBP attitude(p=.002)<br>-EBP skills(p<.001)<br>-EBP knowledge(p=.325)<br>-EBP overall score(p<.001)-<br>-Overall learning satisfaction(p=.687)<br>-Performance of team's research protocol(p=.049) | 1)EBP-COQ [30] |

| | | | | | | | | | | | |
|---|---|---|---|---|---|---|---|---|---|---|---|
| 29† | Ruzafa-Martínez (2023) Spain | nRCT | 1)4th<br>2)295, 143/152 | E:22.29±5.11<br>C:23.22±6.15 | Flipped classroom on EBP<br>-Cultivating a spirit of inquiry within an EBP culture and environment<br>-Asking the burning clinical question in PICOT format<br>-Searching for and Collecting the most relevant best evidence<br>-Critically appraise the evidence<br>-Integrating the best evidence with one's clinical expertise and patient/family preferences<br>-Evaluating outcomes of the practice decision or change based on evidence<br>-Disseminating the outcomes of the EBP decision or change | Flipped classroom<br>-Seminar and laboratories<br>-EBP-eToolkit | 1) 15 wks<br>2) 150 hrs<br>3) 6<br>(40h hours face-to-face 110hours independent student work) | Traditional teaching with face-to-face | E:185->143(22.7%)<br>C:200->152(24.0%)<br>/No/Yes | -EBP attitude(p=.01)<br>-EBP knowledge(p=.188)<br>-EBP skills(p=.019)<br>-EBP competence(p=.003)<br>-Final exam(p=.206) | 1)EBP-COQ [30] |

Cont: Control group; CBL: Computer-based learning; Exp: Experimental group; EBCPG: Evidence-Based Clinical Practice Guideline; EBN: Evidence-Based Nursing; EBP: Evidence-Based Practice; EBP-COQ: Evidence-Based Practice Competency Questionnaire; EBPQ: Evidence-Based Practice Questionnaire; EC for EBP: Essential competencies for Evidence-Based Practice; Hrs: Hours; ITT: Intention-to-treat; nRCT: non-Randomized Controlled Trial; PBL: Problem-based learning; PICO: Participants, Intervention, Comparison, and Outcome; PICOT: Participants, Intervention, Comparison, Outcome, and Time; RCT: Randomized Controlled Trial; Ref: Reference; SD: Standard deviation; TBL: Team-based learning; Wks: Weeks, †: Meta-analysis included.
